# Evaluation of comorbid psychological disorders in functional gastrointestinal disorders patients by vibraimage technology: protocol of a prospective, single-center trial

**DOI:** 10.3389/fmed.2024.1452187

**Published:** 2024-08-30

**Authors:** Chen Yang, Lyu Chengzhen, Yang Daiyu, Tang Hao, Gong Liang, Li Jian, Li Xiaoqing, Wu Dong

**Affiliations:** ^1^State Key Laboratory of Complex Severe and Rare Diseases, Department of Gastroenterology, Peking Union Medical College Hospital, Chinese Academy of Medical Sciences and Peking Union Medical College, Beijing, China; ^2^Chinese Academy of Medical Sciences and Peking Union Medical College, Beijing, China; ^3^State Key Laboratory of Complex Severe and Rare Diseases, Department of Internal Medicine, Peking Union Medical College Hospital, Chinese Academy of Medical Sciences and Peking Union Medical College, Beijing, China; ^4^Beijing Sino Voice Technology Co., Ltd., Beijing, China; ^5^Department of Gastroenterology, The People’s Hospital of Tibetan Autonomous Region, Lhasa, China

**Keywords:** functional gastrointestinal disorders, psychological disorders, vibraimage technology, screening efficacy, therapeutic outcomes

## Abstract

**Introduction:**

Functional gastrointestinal disorders (FGIDs) affect over 40% of individuals globally, and impact the quality of life. A significant portion of FGIDs patients comorbids with anxiety and depression. Traditional screening tools for psychological disorders may lack comprehensiveness. Vibraimage technology currently enables non-contact, objective analysis of psychological indicators through high-frame-rate cameras and computer analysis of micro-movements. Therefore, this study aims to (1) explore the use of vibraimage technology as a non-contact objective method to assess the psychological status of FGIDs patients, comparing this technology with the Hospital Anxiety and Depression Scale (HADS) to evaluate its screening efficacy, and (2) observe the therapeutic outcomes of FGIDs patients with or without comorbid psychological disorders after the same conventional treatment.

**Methods:**

This is a prospective, single-center observational trial. 276 FGIDs outpatients who visit Peking Union Medical College Hospital will be evaluated simultaneously by HADS and vibraimage technology, then to evaluate the screen efficacy of this technology. The patients will be allocated into two groups (those with or without psychological disorders). The primary endpoint is the overall rate of improvement, specifically referring to the proportion of patients who achieved Likert scores greater than or equal to 4. The secondary endpoints encompass evaluating whether there is a reduction of more than 50% in symptom evaluation scores such as IBS-SSS. Additionally, the study will assess changes in health status and quality of life using SF-36 questionnaires and the patients’ satisfaction with treatment. Furthermore, psychological status will be reassessed by vibraimage technology and HADS after treatment to evaluate the effect of combined psychological factors on FGIDs treatment.

## 1 Introduction

Functional gastrointestinal disorders (FGIDs), also known as disorders of gut–brain interaction (DGBI), manifest as a variety of gastrointestinal symptoms such as nausea, vomiting, abdominal pain, bloating, diarrhea, and constipation, which have been excluded organic lesions through objective examinations. Functional dyspepsia, irritable bowel syndrome and functional constipation are the most common FGIDs in clinical practice (1). The pathophysiological mechanisms of FGIDs are complex, involving abnormalities in gut–brain interaction, dysbiosis of intestinal flora, alterations in mucosal immune function, visceral hypersensitivity and gastrointestinal dysmotility (2). A large-scale epidemiological study showed over 40% of individuals worldwide suffered from FGIDs, which significantly impacting the quality of life (3). Recent studies have indicated that approximately 50% of FGIDs patients also experience comorbid anxiety and depression (4–6), which leads to a poor response to conventional treatments. As a result, these patients often require the use of neuromodulators in conjunction with cognitive-behavioral therapy to achieve clinical relief. In accordance with the Rome Foundation’s definitional guidelines, neuromodulators are divided into central and peripheral types, with central agents such as antidepressants, antipsychotics, and buspirone, and peripheral agents including serotonergic drugs, chloride channel modulators, and α2δ (delta) ligands (7). Most gastrointestinal specialists may lack a background in psychology. And up to now, there are no rapid and effective screening tools for identifying FGIDs patients comorbid with psychological disorders, resulting to recurrent visits, cost issues and substantial healthcare resource utilization (3, 8, 9).

Various scales based on psychological questionnaires may assess and screen mental health disorders, such as the Hospital Anxiety and Depression Scale (HADS), Patient Health Questionnaire-9 (PHQ-9), and Generalized Anxiety Disorder-7 (GAD-7). Among these, as for the simplicity, short time to assess, good reliability and validity, HADS has become a widely used self-assessment questionnaire to evaluate anxiety and depression (10). However, questionnaire-based screening entails some subjectivity and may not encompass other psychological status like anger and fear (11). Structured psychological interviews based on the diagnostic and statistical manual of mental disorders (DSM) offer a comprehensive evaluation of the patient’s psychological states but are relatively time-consuming and unsuitable as an initial screening tool (12).

The fact that physiological reflex movements reflect emotional and psychophysiological states has long been recognized (13). Greater uniformity, symmetry, and fluidity in movement are indicative of a normal and positive psychophysiological state, while irregularities, asymmetries, and lack of fluidity in movement suggest the presence of some pathological condition. Based on this, vibraimage technology studies the physiological features of reflex movements of the head at a micro-vibration level, beyond what is visible to the naked eye (14). This advanced, non-contact, non-interactive assessment method overcomes the subjectivity of traditional questionnaires. Utilizing high-frame-rate cameras, it captures micro-vibration movements of the head, neck, and facial muscles, which are then converted into pixel motion through computer analysis. This process captures three-dimensional trajectories and calculates the amplitude and frequency of vibrations for each pixel. Video motion magnification algorithms and comparisons with a basic feature model library enable the analysis of potential micro-expressions and various psychological indicators. This innovative technology is particularly useful in clinical settings, where it can monitor patient responses and detect subtle signs of distress or discomfort that might not be evident through standard observational methods (15–17).

This study aims to evaluate the psychological status of FGIDs patients using vibraimage technology and compare it with HADS to assess the clinical screening efficacy of this technology. By doing so, the study seeks to provide a theoretical basis for the significance of comorbid psychological disorders in FGIDs patients and the necessary of early multidisciplinary collaborative treatment for this patient population, ultimately improving their overall health outcomes.

## 2 Methods

### 2.1 Design

**Figure 1:**
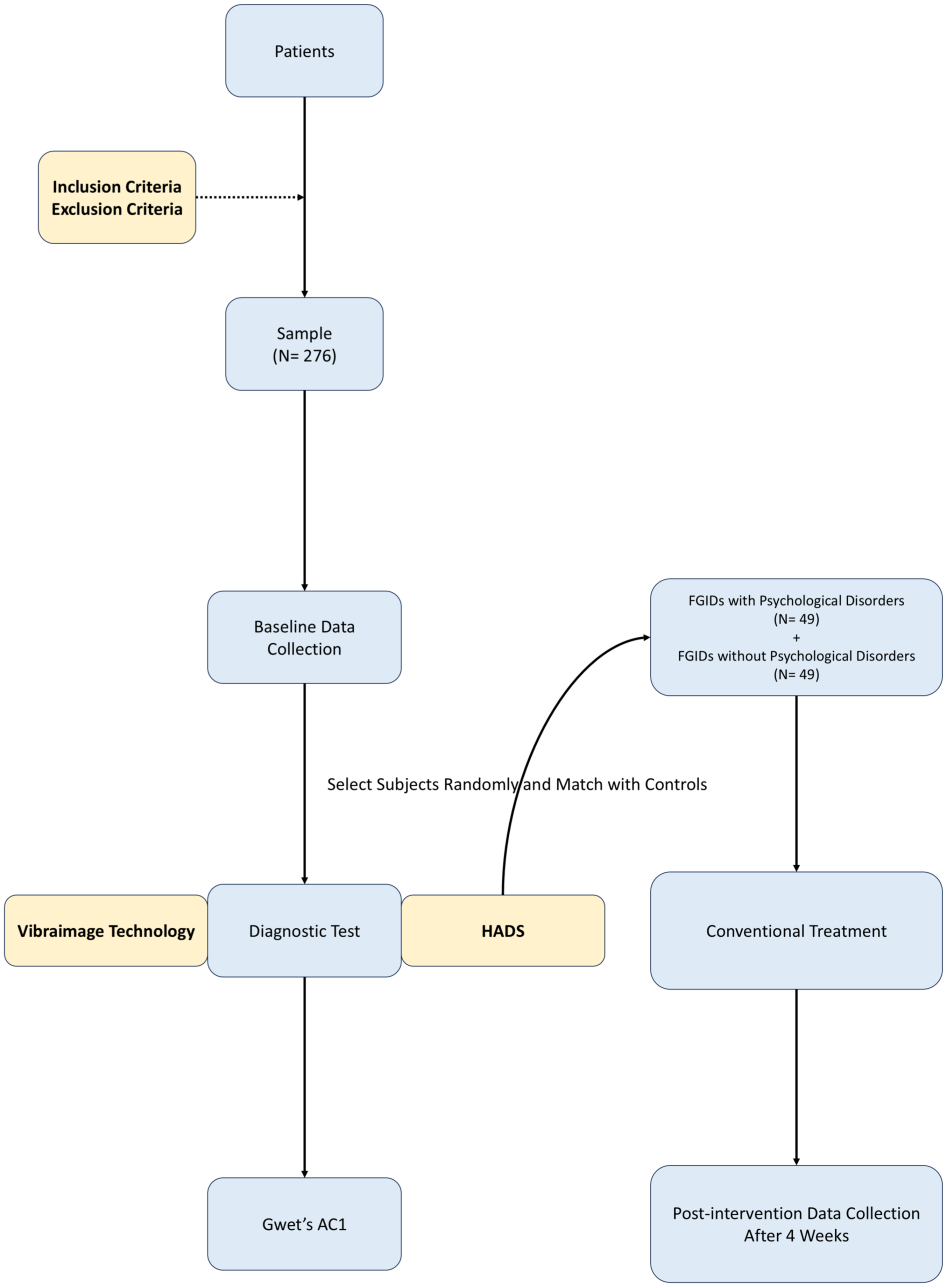
Flowchart of the study process. HADS, hospital anxiety and depression scale; Gwet’s AC1, Gwet’s agreement coefficient 1.

This is a prospective, observational, single-center trial. The patients with FGIDs will be continuously enrolled in the outpatient clinic by experienced physicians from the Department of Gastroenterology at Peking Union Medical College Hospital, who will strictly adhere to the guidelines. The diagnosis of FGIDs adheres to the diagnostic criteria outlined in Rome IV (1), encompassing primarily functional dyspepsia, irritable bowel syndrome, functional constipation, and other FGIDs. The diagnostic criteria for the aforementioned diseases are elaborated in the inclusion criteria. Baseline data including age, sex, marital status, employment status, body mass index (BMI), FGIDs subclasses, scores of symptoms and quality of life were recorded. The simultaneous evaluation of HADS and vibraimage technology will be conducted. The clinical screening efficacy of vibraimage technology will be assessed by comparing with HADS, which serves as the gold standard. The patients will be divided into two groups according to HADS, that is, FGIDs with or without psychological disorders. Both groups will be treated with identical conventional therapies. Post-treatment assessments of symptom questionnaire, HADS and vibraimage technology outcomes will be followed up, which may show the influence of psychological factors and provide a theoretical basis for the significance of identification and treatment of comorbid psychological disorders in FGIDs patients. The trial design is presented in Figure 1, while the subsequent sections provide a comprehensive and detailed description of our trial.

### 2.2 Study population

All patients with FGIDs presenting to Peking Union Medical College Hospital, a tertiary hospital in Beijing, China, will be assessed for eligibility during the appointment, starting from January 2025 and being estimated to be completed in December 2025.

#### 2.2.1 Inclusion criteria

1. Age 18–80 years.
2. The patients diagnosed with FGIDs during outpatient visits to the Department of Gastroenterology at Peking Union Medical College Hospital and who will provide consent for follow-up treatment and care. The study will primarily enroll patients who meet the Rome IV criteria (18) for functional dyspepsia, irritable bowel syndrome, functional constipation, and other FGIDs. Other FGIDs encompass chronic diarrhea, centrally mediated abdominal pain syndrome, functional esophageal disease, and functional anorectal disease.
3. The patients must provide consent for the collection of facial video and completion of HADS psychological self-assessment.
4. The patients must consent to undergo conventional treatment for FGIDs.
5. The patients are able to sign the informed consent.

#### 2.2.2 Exclusion criteria

The patients who fulfill any of the following criteria will be excluded:

1. Age under 18 years old or over 80 years old.
2. The patients presenting with celiac disease, inflammatory bowel disease, or other organic gastrointestinal disorders are typically diagnosed based on evident structural abnormalities or pathological changes. This comprehensive evaluation includes a detailed medical history, thorough physical examination, and auxiliary diagnostic tests.
3. The patients presenting with secondary gastrointestinal motility disorders due to primary diseases such as cholecystitis, pancreatitis, hyperthyroidism, diabetes, chronic renal insufficiency, and neurological diseases.
4. The patients who are reluctant to seek medical treatment and attend regular follow-up appointments at our hospital.
5. The patients are either pregnant or lactating.
6. The patients with severe comorbidities that impact medication management, such as severe hepatic insufficiency, renal failure, and congestive heart failure. The exclusion of such patients is a precautionary measure to avoid potential adverse drug reactions and to maintain the efficacy and safety of the study’s intervention.
7. The current medication regimen includes antipsychotics, spasmolytics, antidiarrheals, probiotics, antibiotics, and other pharmacological agents utilized for the management of abdominal pain, intestinal peristalsis disorders, and gastrointestinal motility disturbances.

#### 2.2.3 Withdrawal criteria

If a participant withdraws from the study based on predefined withdrawal criteria, any data collected from that participant up to the point of withdrawal will be excluded from subsequent analyses. This approach ensures the integrity of the study results and maintains consistency in data analysis.

1. Subjective inclination towards study withdrawal due to various considerations subsequent to enrollment.
2. The patients are unable to successfully complete the vibraimage technology process or undergo HADS psychological self-assessment in this study.

### 2.3 Baseline data collection

Vibraimage data specialist is responsible for collecting and analyzing the patient’s vibraimage technology data, while the HADS assessment specialist will handle the general information collection and analysis of the patient’s HADS self-rating data. Both specialists are unaware of each other’s datasets. The physicians will solely administer conventional treatments based on FGIDs diagnostic criteria, without any knowledge of the results obtained from vibraimage technology or HADS questionnaires.

The following information will be collected: demographic data (age, gender, height, weight), marital and childbearing status, occupation and education level; disease-related details including course of illness, diagnosis and classification of FGIDs, treatment process, and dietary habits. The FGIDs symptoms will be generally assessed by5-point Likert score. Patients diagnosed with irritable bowel syndrome will be evaluated based on their IBS Severity Score (IBS-SSS) (19); patients with functional dyspepsia, as well as those with constipation, will be assessed using the relevant symptom scores (20, 21); while patients with other functional disorders will be evaluated through the Gastrointestinal Symptom Severity Index (GISSI) (22). Baseline questionnaires will be administered to assess anxiety and depression levels using the Hospital Anxiety and Depression Scale (HADS), and general health-related quality of life using the Health Status Questionnaire (SF-36) (23).

### 2.4 The principles and implementation of vibraimage technology

The vibraimage technology in this study is supported by Beijing Sinovoice Technology Co., Ltd.

The imaging principle, vibration extraction method, and vibration visualization method involved in the vibration measurement method based on machine vision have been thoroughly investigated (24–27). Video image amplification technology is utilized to extract and amplify minute vibrations in the video, while vibration visualization is achieved through video reconstruction for enhanced amplification of video images. The primary focus of this research is to directly extract structural vibration signals by analyzing local phase changes in the image. Spatial filtering (24) is applied to calculate local phase and amplitude information for each pixel in every frame of the video image. Phase information indirectly represents subtle motion within the video, which can be amplified by processing this phase information using Fourier transform’s time-shift property (25) that transforms position information into frequency domain phase information. By calculating phase contour videos at different scales, the phase difference signal for each pixel position within these contour videos can be extracted as a vibration signal. The averaged frequency spectrum of all phase difference signals serves as the frequency spectrum for the vibration signal, leveraging correlations between image frequency domain information and motion (26). Furthermore, denoising techniques (27) are employed on local amplitudes to enhance measurement accuracy.

**Figure 2:**
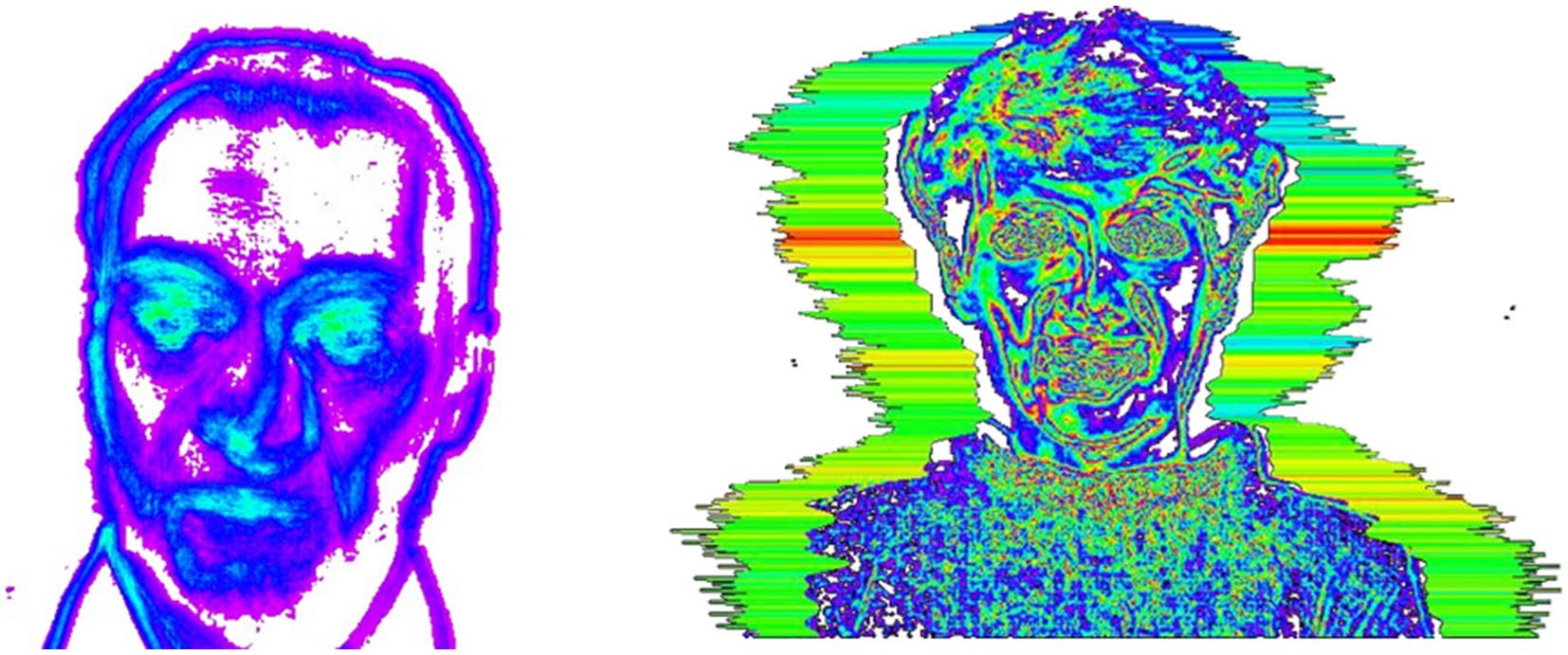
Human micro-vibration images.

Human micromovements, also known as vibrations, can be measured using standard digital, network or television cameras and image processing technologies. These movements are closely linked to the human vestibulo-emotional reflexes (VER) and can reflect emotional states and personality traits (28). By detecting three-dimensional (3D) head and neck movements over several video frames, fluctuations in emotions can be identified (29). As depicted in Figure 2, each pixel of a video image represents a vibration parameter such as frequency or amplitude. The visualization of these parameters is called an external vibration image (30) which resembles a vibratory halo around the person’s head. Generally, the color of this image corresponds to the vibration frequency while its size reflects the amplitude. This mapping of micro-changes to individuals’ psychological states allows for the development of a video-based psychological assessment method. Currently, this technology can give assessment reports of patients’ psychological status, for example, whether they have anxiety or depression status. The primary application of this technology lies in the realm of public safety, encompassing lie detection and human-computer interaction systems (31–34). Furthermore, it holds significant potential for clinical disease diagnosis, particularly in cases related to depression (35). The patients diagnosed with FGIDs will be instructed to assume an upright position, facing the camera, for approximately three minutes of video recording.

### 2.5 Conventional treatment

Patients enrolled in the study with FGIDs will receive conventional specialty care in gastroenterology. The clinician needs to determine which symptom features are dominant, and which treatment (s) are most likely to lead to improvement. Certain patients may find simple, cost-effective interventions such as laxatives or anti-diarrheal medicine (2). Treatment principles for patients with the same subtype of FGIDs are consistent, but actual treatment plans may vary slightly based on individual patient circumstances. The conventional treatment in this study does not encompass the utilization of psychotherapeutic drugs and psychotherapy.

### 2.6 Outcome measures

#### 2.6.1 The evaluation metric for the diagnostic test

Primary Outcome: Sensitivity and specificity of vibraimage technology compared to HADS as the gold standard.

Secondary Outcomes: Positive predictive value (PPV), negative predictive value (NPV), and overall accuracy of vibraimage technology.

#### 2.6.2 The assessment of patient prognosis and satisfaction

The patients will be divided into two groups according to HADS, that is with or without psychological disorder group. The patients in both groups will receive the conventional treatment, without any additional interventions beyond the standard care for FGIDs. The treatment effect questionnaire will be administered after 4 weeks. Overall improvement will be evaluated using a 5-point Likert scale (36), where patients will be prompted to complete the following statement: “How does my gastrointestinal condition compare to before I commenced treatment at the clinic?” The response options include: deteriorated, slightly deteriorated, unchanged, slightly improved, and significantly improved. The HADS and vibraimage technology will also be systematically evaluated.

Primary endpoint: the overall rate of improvement, specifically referring to the proportion of patients who achieved Likert scores greater than or equal to 4.

Secondary endpoint: HADS and vibraimage technology outcomes, along with the improvement degree of FGIDs symptom scores such as IBS-SSS, quality of life by SF-36 questionnaires, and the patients’ satisfaction with treatment.

### 2.7 Sample size calculation

The sample size required for a trial comparing the efficacy of HADS and vibraimage technology in detecting comorbid psychological disorders in FGIDs patients is calculated here. Based on a literature search, it is evident that the prevalence of psychological disorders in FGIDs is 50%. Referring to previous methodological studies (22), we assume a sensitivity of 80% for vibraimage technology, a significance level (*α*) of 0.05, a test power (1 − *β*) of 0.8, and a margin error of 7%. Consulting relevant tables, we determine that the target sample size should be 251 individuals with an anticipated attrition rate of 10%, resulting in a final target sample size of 276 patients.

In addition, we conduct an analysis to determine the required sample size for the post-treatment follow-up experimental process in patients with FGIDs. Based on a literature review, the overall rate of improvement (P1) in FGIDs patients after receiving conventional treatment regimen is reported as 57% (37). We expect that patients with both FGIDs and psychological disorders will have an overall rate of improvement (P2) following conventional treatment at a ratio of 1: 2 compared to P1. The sample allocation between these two groups is set at 1: 1, with a test level *α* of 0.05, test efficacy (1 − *β*) of 0.8, and a loss rate of 10%. Through analysis using PASS software (V21.0.3), the target sample size is determined to be 98 individuals.

The specific distribution scheme of the sample can be observed in Figure 1.

### 2.8 Data management

Suitably trained doctors will conduct face-to-face interviews with each patient to facilitate the completion of a questionnaire prior to undergoing assessments. The same doctors will then provide ongoing assistance to patients during subsequent visits, ensuring the avoidance of potential biases.

The data analysis for this study will be conducted on the Intranet computer, and the research and analysis dataset will be established based on the Intranet data analysis platform. Data sorting and analysis will be conducted by authorized researchers who are blinded to the assignment of study groups within the unit. All processes of data processing and analysis are documented, while preserving the original dataset.

### 2.9 Descriptive statistics

The consistency analysis is conducted based on Gwet’s Agreement Coefficient 1 (AC1, 38) to assess the concordance between vibraimage technology and HADS detection results, as well as to determine the optimal threshold for vibraimage technology. Specifically, a HADS-A score of 8 or higher indicates anxiety, while a HADS-D score of 8 or higher suggests depression (10).

The measurement data that conform to a normal distribution are represented by *x̄* ± *s*, while the ones that do not conform are represented by the median (interquartile range). The difference between the two groups of measurement data following a normal distribution is assessed using a student *t*-test, whereas the difference between the two groups of measurement data not following a normal distribution is assessed using Mann–Whitney U test. The difference between the two groups is evaluated using *χ*^2^ test. Oneway-ANOVA analysis is employed for group comparisons. Pearson correlation analysis is used for measurement data with a normal distribution, and Spearman correlation analysis is used for measurement data without a normal distribution.

In the process of conducting binary logistic regression analysis to determine whether patients with FGIDs are also affected by psychological disorders, we will continuously enhance the model’s goodness of fit by eliminating irrelevant independent variables. Subsequently, a diagnostic model with high diagnostic efficiency will be derived. Finally, a Receiver Operating Characteristic (ROC) curve will be constructed based on the performance of sensitivity and specificity at various thresholds. This analysis will aid in identifying the optimal diagnostic test threshold and in evaluating the accuracy, sensitivity, and specificity of the test.

Variables with a *p* value <0.05 are considered statistically significant. The statistical analysis will be conducted by a medically trained personnel who will remain blinded to the assignment of study groups. The data statistics in our study will be conducted using paper questionnaires. In the event of missing items, we will either discard the questionnaire or, if necessary, employ imputation techniques to fill in the missing values.

### 2.10 Trial management

This study will establish a dedicated team for data analysis and quality control to ensure the integrity, authenticity, and logical consistency of the data. Cases will be rigorously screened and excluded based on strict inclusion criteria, while the quality of the data will be thoroughly examined. In accordance with GCP requirements, comprehensive management of source data recording, sorting, and analysis will be implemented to guarantee that research findings are accurate, reliable, complete, and truthful. Moreover, an expert group comprising professionals in epidemiology, gastroenterology, and other relevant fields will be formed to conduct comprehensive quality control over data analysis and interpretation of study results.

### 2.11 Confidentiality

The documents, reports, and information pertaining to the subject of the study are strictly maintained in a confidential manner. Data analysis will be conducted on an intranet computer system, ensuring that the original data remains isolated from external networks to prevent any potential data breaches. The research report shall not include any identifying information about the subjects, such as their names or ID numbers. Each study participant is assigned a unique study ID number. Researchers seeking access to the identities of research subjects must first obtain approval from the ethics committee.

### 2.12 Patients and public involvement

The involvement of patients and/or the public in the trial’s design, conduct, reporting, or dissemination plans was not incorporated.

## 3 Strengths and limitations of the study

1. This is a prospective, single-center trial, which allows for the examination of progression of FGIDs patients over time and ensures consistency in the study protocol and data collection. But at the same time, it may affect the generalizability of the findings and not be applicable to other populations or healthcare settings.
2. Vibraimage technology is a novel approach to objectively assess the psychological status of FGIDs patients.
3. The assessment of gastrointestinal and mental health symptoms may be subject to biases, such as the patient’s perception and memory of their symptoms.
4. This study has a long follow-up period, which may lead to dropouts and data loss.

## 4 Ethics and dissemination

The research ethics committee of the Peking Union Medical College Hospital has granted ethical approval (I-23PJ974) for this study. Ethical approval was obtained from a single site, as all procedures will be conducted at the Peking Union Medical College Hospital. This protocol has been approved and registered with the Chinese Clinical Trial Register (ChiCTR2400080796). The findings of this trial will be published in an open-access format and disseminated among gastrointestinal physicians.
